# Internet use and health status among older adults: The mediating role of social participation

**DOI:** 10.3389/fpubh.2022.1072398

**Published:** 2022-11-25

**Authors:** Bin Hou, Yumei Li, Haixia Wang

**Affiliations:** ^1^School of Cultural Tourism and Public Administration, Fujian Normal University, Fuzhou, China; ^2^Department of Social and Behavioral Sciences, City University of Hong Kong, Hong Kong, Hong Kong SAR, China; ^3^School of Social and Behavioral Sciences, Nanjing University, Nanjing, China

**Keywords:** internet use, health status, social participation, older adults, aging

## Abstract

**Background:**

The acceleration of population aging and the arrival of the informatization make more and more older adults use the Internet, and its use is having an impact on their health. However, the relationship between internet use and the health of older adults and the mechanism of the effect of internet use on the health are not very clear.

**Methods:**

Multiple linear regression models to explore the correlation between internet use and health status in the 3,141 individuals aged ≥60 years were used. Propensity score matching (PSM) method was used to test the robustness of the regression results. In addition, sequential recursive models was used to examine the mediating effect of social participation on the relationship between internet use and health status.

**Results and discussion:**

We found a significant positive relationship between internet use and health status, and social participation mediated the relationship between internet use and health status. In addition, the effect of internet use on health status was different among older adults in rural and urban areas.

**Conclusions:**

The development and application of internet products adapted to the development of an aging society should be accelerated to meet their needs for continued socialization. The forms and activities of social participation for the elderly groups should be enriched and public service internet usage training seminars should be conducted to improve internet skills.

## Introduction

Relevant statistics show that in 2018, for the first time, the number of older people (over 60 years old) in China exceeded the number of people under the age of 15. In 2019, the number of people aged 60 and older in China was 253 million, accounting for 18.1% of the total population and 12.6% of the population aged 65 and over. With the rising trend of population aging, the health problems of the elderly population cannot be ignored (1, 2). Good health not only enables the elderly to enjoy a good life in their old age but also helps to reduce the burden of China's social retirement, alleviate the pressure of insufficient medical resources, and provide a guarantee of economic development. Therefore, it is an important task for China to improve the health of elderly individuals, ensure their wellbeing and enable them to achieve “healthy aging”.

Along with aging comes high-speed informatization. The popularity of the internet has changed people's lifestyles, and an increasing number of people enjoy convenient information delivery and rich life services through the internet (3, 4). In June 2020, the proportion of Chinese internet users reached 940 million, and the internet penetration rate reached 67.0%. Meanwhile, the proportion of older internet users is also increasing. The proportion of internet users aged 60 and above rose from 6.7% in March to 10.3% in June. The use of the internet has also continued to change the daily lifestyle, behavior and attitude of the elderly population (5, 6). Scholars have proposed socialization theory, which suggests that socialization can be divided into four parts: basic socialization, anticipatory socialization, developmental socialization and resocialization. Resocialization is the idea that individuals still need to adapt to society and engage in self-improvement through continuous learning in old age. For older adults, resocialization is also key to maintaining health and achieving longevity (7). In the context of information technology, older adults need to continuously learn new skills and actively adapt to new lifestyles through the application of the internet to facilitate their successful resocialization. The use of the internet as a new skill and lifestyle can play an important role in the resocialization process of older adults, which in turn can contribute to the improvement of their health status. Therefore, in the context of aging, this paper examines the impact of internet use on the health status of older adults using data from the CGSS 2018 to analyze the mediating effect mechanism of social participation and to examine the heterogeneity of the effect of the internet on the rural and urban elderly populations.

## Literature review and hypotheses

With increasing age, individuals suffer from significant changes in their physical, psychological and social roles. When entering old age, people experience a significant increase in physical and psychological morbidity, making older adults the most vulnerable group in terms of health (8). Previous studies on factors influencing the health of older adults have addressed factors such as gender, age, educational attainment, marital status, physical activity, socioeconomic status, and participation in social insurance. Older male adults have better health status than older female adults (9). Age is inversely related to the health status of older adults to some extent (10). Improvement in education leads to improvement in the health status of older adults (11). Older married adults are healthier than old unmarried and widowed adults (12). Older adults who participate in regular physical activity have higher health indices (13). Higher socioeconomic status significantly affects the health of older adults (14). Older adults who participate in health insurance have higher self-rated health scores (15). Pensions also significantly improve the mental health of older adults (16). Therefore, we added these factors as control variables in the regression models that followed.

Previous studies have shown that internet use significantly affects the health status of individuals. However, scholarly research on this issue presents two contrasting views. Some studies argue that internet use is beneficial to the health status of individuals (17–20). Specifically, this health-promoting effect is reflected in three aspects. First, internet use helps individuals have sufficient health information for better self-management. Scholars have found that older adults can significantly improve their health by acquiring health-related knowledge through the internet (21). Internet use breaks the monopoly of doctors on professional information, alleviates information asymmetry between doctors and patients, and facilitates residents to better manage their health (22). In addition, the researchers assessed the relationship between internet use, socioeconomic status and social support and self-rated health, and the results showed that internet health information users had better health status than users who did not use online health information as a reference (23). Second, as a tool for communication, the internet can alleviate users' loneliness, depression, and anxiety and enhance users' health. Researchers used the U.S. Health and Retirement Survey (HRS) data from 2002 to 2008 in the United States to examine the effect of internet use on depression among older adults and found that compared to not using the internet, when using the internet, older adults were 33% less likely to be depressed (24). Using a sample of 245 U.S. adults, researchers examined the psychological determinants of internet health information use with structural equation modeling and showed that the internet can reduce stress and lower levels of depression and loneliness (25). Third, internet use can alleviate and prevent a variety of health disorders. Residents who use the internet regularly are less likely to suffer from depression (26), and internet use also reduces anxiety levels in patients with cardiomyopathy (27). Other studies have suggested that the internet can lead to health deterioration due to addiction. Researchers have found that increased internet use may predispose people to internet addiction and online gaming, which in turn increases psychological anxiety, depression and social fear (28–31).

Based on the above discussion, we propose the following hypotheses.

Hypothesis 1: Internet use significantly affects the health status of older adults.

Social participation is an important way for older individuals to reach out to society, engage in interpersonal interactions, and maintain social connections, and it is an important intervention to promote active and healthy aging, which can effectively prevent individuals from being closed off to others (32). It enables older adults to participate in the political life of the community (33), work for pay (34), and socialize with others (35). Social participation has an impact on older adults' cognitive ability as they age, and the higher the ability to participate socially is, the stronger the cognitive ability (36). Social-emotional choice theory points out that social participation requires a certain cost investment, and members who engage in social participation are bound to consider cost-benefit issues (37). Offline social participation decreases as the physical and mental ability of elderly individuals decreases. The attributes of the internet can break through the limitations of time and space and reduce the physical ability requirements, which can compensate for the reduced offline social participation due to declining physical ability. Previous research suggests that internet use affects individuals' social participation in different ways. The internet may be an important means for older adults to maintain existing interpersonal relationships and expand their social networks (38). A statistical analysis of data from a large U.S. survey found that internet use increased residents' motivation to participate in community activities (39). Researchers studying internet use among older adults in the Netherlands noted that internet use plays a positive role in increasing older adults' social capital, allowing them to expand or maintain social connections and expand their access to information (40). In addition, social engagement can have a significant impact on an individual's health status. Researchers analyzed a sample of 43 neighborhoods in Shanghai across age groups and found that neighborhood environment and social engagement in all samples had a significant positive impact on older adults' health. At the same time, social participation became a mediator of the relationship between the interpersonal environment and older adults. The study also found that social participation mediated the relationship between the interpersonal environment and the health of older adults (41). A survey of 28,895 individuals aged 45–84 years in China found that social participation positively affects health by improving individual mental health effects and increasing individual income effects (42).

On the basis of the above discussion, we propose the following hypothesis:

Hypothesis 2: Social participation mediates the relationship between internet use and older adults' health.

Therefore, this study aimed to explore the relationship between internet use and older adults' health to provide guidance and help improve older adults' health. Meanwhile, to further understand the impact of internet use on the health of elderly individuals and to find ways to improve their health, this study chose social participation as a mediating variable. The findings of this paper facilitate a deeper understanding of the relationship between internet use and residents' health while examining the possible channels through which internet use affects older adults' health from the comprehensive dimension of social participation. This study fills the gaps in existing studies and provides a realistic basis for better guidance on internet use to improve older adults' health.

## Methods

### Data and study sample

This study used data from the CGSS collected by the China Survey and Data Center of Renmin University of China. The data was collected using a stratified sampling method, and the database covers 28 provinces (municipalities and autonomous regions) in China with details about the health status, individual characteristics, and household characteristics of the population. The CGSS 2018 data were used in this study. The population of this study is the elderly population, and the method of screening the study population is to first find the variable of the year of birth of the sample in the questionnaire, then subtract the year of birth from 2018 to obtain the age of the sample, and finally screen out individuals 60 or older. In this study, a total of 3,141 valid sample sizes were obtained after removing the missing values of the independent, dependent and control variables.

### Measures

#### Dependent variable

The dependent variable in this study is the health status of elderly individuals. Self-rated health (SRH) was used to measure health status. The question was “What do you think of your current health status?”. Possible responses were “1 = very unhealthy”, “2 = less healthy”, “3 = fair”, “4 = healthy”, and “5 = very healthy”; the higher the value was, the better the health condition. Self-rated health status is considered to be a simple indicator to measure the health status of the old population and was found to predict future health outcomes (43, 44).

#### Independent variable

The independent variable in this study is internet use. The question used to measure the dependent variable was “In the past year, how much did you use the internet?” Responses were “1 = never”, “2 = rarely”, “3 = sometimes”, “4 = often”, and “5 = very often”; options 2 to 5 were combined and coded as “1 = use the internet”; and option 1 was coded as “0 = never use the internet”, making it a binary categorical variable.

#### Mediating variable

The mediating variable in this study is social participation, and according to the definition of social participation in previous studies, economic participation (45), political participation (46), and social participation (36, 47) are included in the measurement of social participation variables in this study. In particular, economic participation is measured by the question “What is your work experience and status?” The responses “currently working in non-farm work”, “currently working in farming”, “have worked in non-farm work”, “currently working in farming,” and “have not worked in non-farm work” were combined to form “1 = participation”. The responses “not currently working”, “have only worked in agriculture”, and “have worked in non-farm work” were combined to form “0 = not participation”. The question to measure political participation was “Did you vote in the last neighborhood council/village council election?” Responses were “1 = participated” and “0 = not participated”. The questions measuring social participation were “In the past year, did you often get together with relatives who do not live together during your free time?” and “In the past year, did you often get together with your friends in your free time?” For these two questions, those who attended parties were coded as “1 = participated”, and those who did not were coded as “0 = did not participated”. These three types of questions were summed to obtain a total score measuring social participation, ranging from 0 to 4. The higher the value was, the higher the social participation.

#### Control variables

We controlled for confounding variables affecting internet use, social participation, and the health status of older adults. These variables included individual characteristics, household characteristics, and social characteristics. Among them, individual characteristics included age, gender (0 = female, 1 = male), education level (0 = illiteracy, 1 = primary school, 2 = junior middle school and high school, 3 = university or more), and marital status (0 = unmarried, divorced or widowed, 1 = married); household characteristics included number of children, cohabitation (0 = not living together, 1 = living together), and housing area. Social characteristics variables included physical exercise (0 = not participating, 1 = participating), residence (0 = rural, 1 = urban), personal socioeconomic status (1 = lower class, 2 = middle class, 3 = upper class), participation in pension insurance status (0 = not participating, 1 = participating), and participation in medical insurance status (0 = not participating, 1 = participating).

### Statistical analysis

Stata 16.0 was used to analyze the effect of internet use on the health status of older adults. First, descriptive statistical analysis was used to describe the data distribution of the independent, dependent, and mediating variables as well as other control variables. Second, a multiple linear regression model was used to analyze the relationship between physical activity and older adults' health status, and then an ordered probit model and propensity score matching method were used to address the endogeneity of the relationship between physical activity and older adults' health status. Finally, we used stepwise regression mediation measures to measure the mediating effect of social participation.

## Results

### Descriptive analysis

Table 1 shows that the mean score for the health status of the sample was 3.164, indicating that the majority of older adults are in a moderate state of health. The mean value of internet use in the sample was 0.334, indicating that less than half of the older adult group used the internet. In addition, 53.33% of the sample was male, 99.33% was married, more subjects had a primary school education level, the average number of children in the sample was 2, 91.72% of the sample lived with their spouses, the housing area was approximately 100.737 square meters, 52.94% participated in physical exercise, and the sample was basically divided equally between rural and urban residences. The socioeconomic status of the sample was at the lower-middle level; 85.23% of the sample participated in pension insurance, and 94.01% participated in medical insurance.

**Table 1:** Sample characteristics.

**Variables**		**Variable definitions**	**Mean**	**SD**
Dependent variable	Health status	Continuous variable, ranging from 1 to 5	3.164	1.046
Independent variable	Internet use	Categorical variable, 0 = never use the internet, 1 = use the internet	0.334	0.472
Mediating variable	Social participation	Continuous variable, ranging from 0 to 4	2.417	0.978
Individual characteristics	Age	Continuous variable	68.146	6.598
	Gender	categorical variable, 0 = female, 1 = male	0.533	0.499
	Marital status	Categorical variable, 0 = unmarried, divorced or widowed, 1 = married	0.993	0.082
	Education level	Categorical variable, 0 = illiteracy, 1 = primary school, 2 = junior middle school and high school, 3 = university or more	1.327	0.909
Household characteristics	Number of children	Continuous variable	2.229	1.267
	Cohabitation	Categorical variable, 0 = not living together, 1 = living together	0.917	0.276
	Housing area	Continuous variable	100.737	56.493
Social characteristics	Physical exercise	Categorical variable, 0 = not participating, 1 = participating	0.529	0.499
	Residence	Categorical variable, 0 = rural, 1 = urban	0.493	0.500
	Personal socioeconomic status	Categorical variable, 1 = lower class, 2 = middle class, 3 = upper class	1.516	0.618
	Participation in pension insurance status	Categorical variable, 0 = not participating, 1 = participating	0.852	0.355
	Participation in medical insurance status	Categorical variable, 0 = not participating, 1 = participating	0.940	0.237

### Impact of physical exercise on health status

This study used multiple linear regression to analyze the effects of internet use on the health status of older adults, and Table 2 demonstrates the specific regression results by gradually putting in individual characteristic variables, family characteristic variables, and social characteristic variables.

**Table 2:** Regression analysis of Internet use on health status of older adults.

	**Model 1**	**Model 2**	**Model 3**	**Model 4**
	**Health status**	**Health status**	**Health status**	**Health status**
Internet use	0.418*** (0.039)	0.323*** (0.045)	0.331*** (0.045)	0.257*** (0.046)
Age		−0.000** (0.000)	−0.000 (0.000)	−0.000 (0.000)
**Gender (female)**		
Male		0.082** (0.038)	0.074* (0.038)	0.092** (0.037)
**Marital status (unmarried, divorced, or widowed)**	
Married		0.230 (0.224)	0.310 (0.232)	0.214 (0.229)
**Education level (illiteracy)**	
Primary school		0.106** (0.052)	0.101* (0.052)	0.066 (0.051)
Junior middle school and high school		0.144*** (0.053)	0.149*** (0.053)	0.048 (0.055)
University or more		0.348*** (0.086)	0.339*** (0.086)	0.153* (0.090)
Number of children			−0.033** (0.017)	−0.019 (0.017)
**Cohabitation (not living together)**				
Living together			−0.078 (0.069)	−0.068 (0.068)
Housing area			0.002*** (0.000)	0.002*** (0.000)
**Physical exercise (not participating)**	
Participating				0.233*** (0.040)
**Residence (rural)**		
Urban				0.050 (0.047)
**Personal socioeconomic status (lower class)**	
Middle class				0.269*** (0.038)
Upper class				0.373*** (0.076)
**Participation in pension insurance status (not participating)**
Participating				−0.074 (0.054)
**Participation in medical insurance status (not participating)**
Participating				−0.025 (0.080)
Constant term	3.024*** (0.022)	2.869*** (0.243)	2.670*** (0.247)	2.690*** (0.254)
N	3,141	3,141	3,141	3,141
R^2^	0.036	0.045	0.053	0.086

*P < 0.10, **P < 0.05, ***P < 0.01. Values in brackets are standard errors.

The regression results from Model 1 reveal that internet use significantly and positively affects the health status of older adults at the 1% significance level. After adding individual characteristic variables, family characteristic variables and social characteristic variables in turn, the regression coefficients of internet use are 0.323, 0.331, and 0.257 shown in Model 2, Model 3, and Model 4, respectively, and they are significant and positive at the 1% significance level, which indicates that internet use has a significant effect on the health status of elderly individuals. The above results support the conclusion that internet use can enhance the health of individuals.

In addition, Model 4 shows the results of the effects of other control variables on the health status of elderly individuals. Gender positively influenced the health level of older adults at the 5% significance level, with older male adults having a higher health status than older female adults. The group with an education level of university or more positively influenced the health of elderly individuals at the 10% significance level, indicating that the higher the education level was, the better the health status. Housing area positively influenced the health of the elderly populations at the 1% significance level. Physical exercise significantly and positively affected the health of elderly individuals at the 1% significance level, and participation in physical exercise can make the elderly healthier. Socioeconomic status positively affects the health of elderly individuals at the 1% significance level, indicating that the higher the socioeconomic status is, the better the health status.

### Robustness test

#### Ordered probit model

The dependent variable of this study, the health status of elderly individuals, takes values from 1 to 5, which can be considered discrete and ordered variables, so we can construct an ordered probit model to test the robustness of the model results in Table 2 to prove the credibility of the regression results in Table 2. Table 3 shows the results of the robustness test, from which we find that the effects of internet use on the health status of elderly individuals are positively correlated at the 1% significance level, which indicates the robustness of the results of this study.

**Table 3:** Robustness test results.

**Variables**	**Model 1**	**Model 2**	**Model 3**	**Model 4**
Internet use	0.416*** (0.040)	0.322*** (0.046)	0.332*** (0.047)	0.263*** (0.049)
Control variables	No control	Individual variables	Individual and household variables	All control variables
N	3,141	3,141	3,141	3,141
LR statistic	107.86***	139.21***	163.25***	275.41***
Pseudo R^2^	0.012	0.016	0.018	0.031

*P < 0.10, **P < 0.05, ***P < 0.01. Values in brackets are standard errors.

#### Propensity score matching

Because of confounding factors that affect both internet use and health status of older adults, sample selection bias was introduced into our results. Therefore, we used propensity score matching to correct the regression results. First, considering that the independent variable used for propensity score matching was a dummy variable, the samples were divided into two groups: the group of participants that participated in internet use and the group that did not. Second, we matched the samples according to the following procedure: (1) We condensed the control variables into an index to calculate the likelihood of each sample belonging to the experimental group. (2) We used five matching methods (1-to-1 nearest neighbor, 1-to-4 nearest neighbor, radius, kernel, and local linear regression matching) to match the experimental group and the control group and to classify individuals with similar values into the same groups to ensure that members of groups had similar characteristics. (3) We calculated the average treatment effect (ATT) on the participants, representing the difference in health status between the experimental and control groups.

Table 4 presents the ATT calculated using different matching methods. The ATT values were >0 in all the models and were statistically significant, indicating that results that internet use has a significant positive impact on the health status of older adults are robust.

**Table 4:** Propensity score matching results (ATT).

		**ATT**	**SE**	**t**
**Dependent variable: Mental health**				
Nearest neighbor matching	1 to 1 matching	3.43***	0.23	4.36
	1 to 4 matching	3.31***	0.25	4.23
	Radius matching	3.67***	0.33	4.19
The whole matching	Kernel matching	3.19***	0.37	4.26
	Local linear regression matching	3.26***	0.27	4.51

***P < 0.01 (t > 2.76), **P < 0.05 (t > 1.96), *P < 0.1 (t > 1.65).

### Testing the mediating effects of social participation

Currently, the mechanism by which internet use affects the health of the population is unclear (20, 24). To effectively reveal this transmission mechanism, the following sequential recursive model was set to test the mediating effect of social participation according to the mediating effect test proposed by Baron and Kenny (48). The first step is to test the effect of internet use on the health status of elderly individuals. If the coefficient of internet use is significant, it indicates that internet use has a significant effect on the health status of elderly individuals. The second step is to test the effect of internet use on social participation as a mediating variable. If the coefficient of internet use is significant, it indicates that internet use can influence the social participation of elderly individuals. The third step is to add the social participation variable to the first step. If the effect of the mediating variable is significant, while the coefficient of internet use decreases or is insignificant relative to the coefficient in the first step, then it indicates that social participation has a partial or even full mediating effect.

Table 5 shows the results of the mediating effects of social participation. In Model 1, internet use positively affects the health status of older adults at the 1% significance level, indicating that internet use enhances the health status of older adults. In Model 2, internet use positively affects older adults' social participation at the 1% significance level, indicating that internet use enhances older adults' social participation. In Model 3, internet use and social participation significantly and positively affect the health status of older adults at the 1% level of significance, and the coefficient of internet use decreases compared to the coefficient of Model 1, which indicates that social participation is one of the mechanisms of action of internet use affecting the health status of older adults, i.e., social participation plays a mediating role in the effect of internet use on the health status of older adults. Internet use can encourage the social participation of older adults, thus enhancing their health. It also confirms the conclusions of existing studies. For example, using survey data from the 2018 China Longitudinal Study of Health and Retirement, the researchers analyzed how Internet use reduced the risk of depressive symptoms through social participation among a sample of 4,645 older adults. The results showed that Internet use improved their health status by increasing social participation (49).

**Table 5:** Internet use and health status of older adults: Mediating role of social participation.

**Variables**	**Model 1**	**Model 2**	**Model 3**
	**Health status**	**Social participation**	**Health status**
Internet use	0.257*** (0.046)	0.137*** (0.043)	0.248*** (0.046)
Social participation			0.065*** (0.019)
Constant term	2.690*** (0.254)	2.452*** (0.238)	2.531*** (0.258)
Control variables	All control variables	All control variables	All control variables
N	3,141	3,141	3,141
R^2^	0.086	0.083	0.089

*P < 0.10, **P < 0.05, ***P < 0.01. Values in brackets are standard errors.

In this study, the mediating effect of social engagement ability was tested again using the KHB method. Table 6 shows the results of the tests of mediating effects. Among them, the total effect of internet use on the health status of the elderly was 0.418, which passed the 1% significance test. The direct effect was 0.394, which passed the 1% significance test, and the indirect effect of internet use on the health status of elderly individuals through social participation ability was 0.024, which passed the 1% significance test, indicating that the mediating effect of social participation ability existed. Therefore, both the stepwise regression mediating effect test and the KBH test in this study found that social participation plays a mediating role between internet use and the health status of elderly individuals. Social participation is the mechanism of action that occurs between internet use and the health status of elderly individuals.

**Table 6:** Mediation test results.

**Mediating variable**	**Effect of type**	**Coefficient**	**Standard error**	**Z**	**P**
Internet use	Total effect	0.418***	0.039	10.81	0.000
	Direct effect	0.394***	0.039	10.11	0.000
	Indirect effect	0.024***	0.006	4.16	0.000

*P < 0.10, **P < 0.05, ***P < 0.01.

### Heterogeneity analysis

The frequency of individual internet use can be influenced by residence (50, 51), and there are differences in the health status of individuals located in different regions (52, 53). Therefore, we asked whether the relationship between internet use and health status differs among older adults located in different regions. We compared the effects of internet use on the mental health of urban and rural older adults (Table 7). The results show that internet use significantly and positively affects the health status of the rural elderly population and the urban elderly population. The marginal effect of internet use on the health status of the rural elderly population was greater than that of the urban elderly population.

**Table 7:** Heterogeneity analysis.

	**Rural elderly**	**Urban elderly**
	**OLS**	**OLS**
Internet use	0.307*** (0.084)	0.220*** (0.054)
Control variables	All control variables	All control variables
N	1,593	1,548
R^2^	0.060	0.091

*P < 0.10, **P < 0.05, ***P < 0.01. Value in brackets are standard errors.

## Discussion

### Internet use can significantly improve the health status of older adults

In this study, we analyzed the relationship between internet use and health status among older adults in China by using the 2018 CGSS data. We identified a significant positive correlation between internet use and health status among older adults in China, which supports H1: Internet use significantly affects the health status of older adults. This conclusion is consistent with previous research findings on the relationship between internet use and health status. For example, in 2008, using data from a survey on the digital divide and quality of life of older adults in Spain, researchers analyzed internet use among the Spanish adult population in two age groups (55–64 and 65–74 years). They found a significant relationship between internet use and poor self-rated health, with internet users having self-rated health status better than that of non-users (54). In addition, researchers selected 3,042 cases of older adults aged 55 years or older in Jiangxi Province, China, in 2018 to analyze the effect of internet use and smartphones on the net effect of internet use and smartphone use on the health of older adults and found that both internet use and smartphone use improved older adults' self-rated health status (55).

### Social participation plays a mediating role in the relationship between internet use and health status

In the present study, we discovered that social participation plays a significant mediating role in the relationship between internet use and health status; that is, social participation is a key mechanism through which internet use affects the health status of older adults, and internet use can improve the health status of older adults by enhancing their social participation. Therefore, H2 is supported. This finding is consistent with previous research findings. For example, researchers analyzed a cross-sectional sample of 11,000 subjects in European countries and found that internet use increased individuals' life satisfaction and decreased their social isolation by increasing their social participation (56). In addition, the researchers analyzed a sample of 240 older adults based on actor network theory and activity theory and found a significant positive relationship between internet use and social participation, with the size of social networks constituting a significant factor in the association between internet use and social participation (57).

### Effect of internet use on health status varies among older adults living in different regions

In the present study, we determined that internet use significantly and positively affects the health status of the rural elderly population and the urban elderly population. The marginal effect of internet use on the health status of the rural elderly population was greater than that of the urban elderly population. A possible explanation for this is that internet penetration is higher in urban areas than in rural areas, so the health promotion effect of internet use on older adults in urban areas becomes equalized. In contrast, in rural areas, internet penetration and use differed between individuals and households, and thus internet use showed differences in health promotion for individuals.

### Limitations

There are some limitations in this study. First, the issues discussed in this paper focus on the difference between use and non-use. In fact, the ability to use internet devices to access information is only a basic skill; the ability to recognize the value of information and quickly access the information needed is more important. However, due to the availability of data, this paper has not been able to examine the impact of internet usage quality on the health of the population, and this issue will be further examined in the future. Second, due to the limitations of public survey data, although this paper reveals the relationship between internet use and the health status of older adults at the individual level, it is unable to explore the psychological mechanisms underlying the interaction between the two at the more microscopic cognitive level. In future research, secondary data studies can be supplemented with quantitative methods such as primary data surveys and experiments to further investigate the impact of internet use on the health status of older adults in different dimensions.

## Conclusions

The results of this study indicate that internet use is significantly and positively correlated with the health status of older adults and that social participation plays a mediating role in the relationship between internet use and health status among older adults. Accordingly, we propose the following suggestions. First, the development and application of internet products adapted to the development of an aging society should be accelerated to enhance the benefits of health improvement for elderly individuals. Internet applications suitable for the elderly individuals should be launched as soon as possible to provide a better experience for them the elderly; further, the functions of terminal devices should be optimized to assist elderly internet users to meet their needs for continued socialization. Second, we enrich the forms and activities of social participation for the elderly groups. The community should provide more internet-related social activities to improve their social participation and encourage users to expand their social participation scope and increase social connection channels, thus improving their health status. Third, public service internet usage training seminars should be conducted to improve internet skills. The findings of this paper indicate that there are urban-rural differences in the effects of internet use on residents' health. Compared with urban areas, the health promotion effect on rural residents is greater. However, non-internet users are mainly concentrated in rural areas, and lack of skills and limited education are important reasons for their lack of access. Therefore, increasing internet skills training is conducive to expanding the population of internet users and enhancing the value of the internet.

## Data availability statement

The original contributions presented in the study are included in the article/supplementary material, further inquiries can be directed to the corresponding author/s.

## Author contributions

BH were responsible for writing and contributed to the conception of the work, data analysis and interpretation, and drafting the article. HW contributed to the data collection, data analysis, and interpretation. YL contributed to give important revising suggestions. All authors contributed to the article and approved the submitted version.

## Funding

This study was supported by Fuzhou Social Science Planning (2021FZB08) awarded to BH.

## Conflict of interest

The authors declare that the research was conducted in the absence of any commercial or financial relationships that could be construed as a potential conflict of interest.

## Publisher's note

All claims expressed in this article are solely those of the authors and do not necessarily represent those of their affiliated organizations, or those of the publisher, the editors and the reviewers. Any product that may be evaluated in this article, or claim that may be made by its manufacturer, is not guaranteed or endorsed by the publisher.

## References

[1] Shu Z Xiao JG Dai XH Han Y Liu YL. Effect of family “upward” intergenerational support on the health of rural elderly in China: Evidence from Chinese Longitudinal Healthy Longevity Survey. PLoS ONE. (2021) 16:e0253131. 10.1371/journal.pone.0253131 34143838 PMC8213075

[2] Yang X Yin DD. The Protective Effect of Caring for Grandchildren on the Mental Health of the Elderly: A Structural Equation Modeling Analysis. Int J Environ Res Public Health. (2022) 19:1255. 10.3390/ijerph19031255 35162285 PMC8834749

[3] Van Deursen A Courtois C Van Dijk J. Internet Skills, Sources of Support, and Benefiting From Internet Use. Int J Hum Comput Interact. (2014) 30:278–90. 10.1080/10447318.2013.858458

[4] Gong Y Zhou JY Ding F. Investigating the demands for mobile internet-based home nursing services for the elderly. J Invest Med. (2022) 70:844–52. 10.1136/jim-2021-002118 34872934 PMC8899476

[5] Peng YI Chan YS. Do Internet Users Lead a Healthier Lifestyle? J Appl Gerontol. (2020) 39:277–84. 10.1177/0733464818785797 29957087

[6] Bianchi C. Exploring how internet services can enhance elderly well-being. J Serv Market. (2021) 35:579–97. 10.1108/JSM-05-2020-0177

[7] Carter LD. Socialization to old-age. Soc Sci. (1976) 51:119–20.

[8] Kong FL Xu LZ Kong M Li SX Zhou CC Zhang JH. Association between socioeconomic status, physical health and need for long-term care among the chinese elderly. Int J Environ Res Public Health. (2019) 16:2124. 10.3390/ijerph16122124 31208088 PMC6617196

[9] Schone BS Weinick RM. Health-related behaviors and the benefits of marriage for elderly persons. Gerontologist. (1998) 38:618–27. 10.1093/geront/38.5.618 9803650

[10] Angelini V Cavapozzi D Corazzini L Paccagnella O. Age, health and life satisfaction among older europeans. Soc Indic Res. (2012) 105:293–308. 10.1007/s11205-011-9882-x 22207782 PMC3228960

[11] Kye B Arenas E Teruel G Rubalcava L. Education, elderly health, and differential population aging in South Korea: A demographic approach. Demogr Res. (2014) 30:753–94. 10.4054/DemRes.2014.30.26

[12] Bos AM Bos AJ. The socio-economic determinants of older people's health in Brazil: the importance of marital status and income. Ageing Soc. (2007) 27:385–405. 10.1017/S0144686X06005472

[13] An S Lee Y Kim JT. The Effect of the Public Exercise Environment on the Physical Activity for the Active Ageing of the Elderly. Indoor Built Environ. (2013) 22:319–31. 10.1177/1420326X1247124635954590

[14] Simsek H Doganay S Budak R Ucku R. Relationship of socioeconomic status with health behaviors and self-perceived health in the elderly: A community-based study, Turkey. Geriatr Gerontol Int. (2014) 14:960–8. 10.1111/ggi.12166 24118995

[15] Liu XT Wong H Liu K. Outcome-based health equity across different social health insurance schemes for the elderly in China. BMC Health Serv Res. (2016) 16:1–12. 10.1186/s12913-016-1261-5 26762146 PMC4712604

[16] Wang F Zheng HT. Do public pensions improve mental wellbeing? Evidence from the new rural society pension insurance program. Int J Environ Res Public Health. (2021) 18:2391. 10.3390/ijerph18052391 33804508 PMC7967743

[17] Mellor D Firth L Moore K. Can the internet improve the well-being of the elderly? Ageing Int. (2008) 32:25–42. 10.1007/s12126-008-9006-3

[18] Erickson J Johnson GM. Internet use and psychological wellness during late adulthood. Canad J Aging. (2011) 30:197–209. 10.1017/S0714980811000109 24650669

[19] Cotten SR Ford G Ford S Hale TM. Internet use and depression among older adults. Comput Human Behav. (2012) 28:496–9. 10.1016/j.chb.2011.10.021

[20] Heo J Chun S Lee S Lee KH Kim J. Internet use and well-being in older adults. Cyberpsychol Behav Soc Netw. (2015) 18:268–72. 10.1089/cyber.2014.0549 25919967

[21] Tse MMY Choi KCY Leung RSW. E-health for older people: The use of technology in health promotion. Cyberpsychol Behav. (2008) 11:475–9. 10.1089/cpb.2007.0151 18721097

[22] Mcmullan M. Patients using the Internet to obtain health information: How this affects the patient-health professional relationship. Patient Educ Couns. (2006) 63:24–8. 10.1016/j.pec.2005.10.006 16406474

[23] Wangberg SC Andreassen HK Prokosch H Santana SM Sorensen T Chronaki CE. Relations between internet use, socio-economic status (SES), social support and subjective health. Health Promot Int. (2008) 23:70–7. 10.1093/heapro/dam039 18083686

[24] Cotten SR Ford G Ford S Hale TM. Internet use and depression among retired older adults in the united states: a longitudinal analysis. J Gerontol B Psychol Sci Soc Sci. (2014) 69:763–71. 10.1093/geronb/gbu018 24671896

[25] Lagoe C Atkin D. Health anxiety in the digital age: An exploration of psychological determinants of online health information seeking. Comput Human Behav. (2015) 52:484–91. 10.1016/j.chb.2015.06.003

[26] Bender JL Radhakrishnan A Diorio C Englesakis M Jadad AR. Can pain be managed through the Internet? A systematic review of randomized controlled trials. Pain. (2011) 152:1740–50. 10.1016/j.pain.2011.02.012 21565446

[27] Minto C Bauce B Calore C Rigato I Folino F Soriani N. Is Internet use associated with anxiety in patients with and at risk for cardiomyopathy? Am Heart J. (2015) 170:87–U123. 10.1016/j.ahj.2015.02.024 26093868

[28] Noh D Kim S. Dysfunctional attitude mediates the relationship between psychopathology and Internet addiction among Korean college students: A cross-sectional observational study. Int J Ment Health Nurs. (2016) 25:588–97. 10.1111/inm.12220

[29] Kitazawa M Yoshimura M Murata M Sato-Fujimoto Y Hitokoto H Mimura M. Associations between problematic Internet use and psychiatric symptoms among university students in Japan. Psychiatry Clin Neurosci. (2018) 72:531–9. 10.1111/pcn.12662 29652105

[30] Sami H Danielle L Lihi D Elena S. The effect of sleep disturbances and internet addiction on suicidal ideation among adolescents in the presence of depressive symptoms. Psychiatry Res. (2018) 267:327–32. 10.1016/j.psychres.2018.03.067 29957549

[31] Ismail N Tajjudin AI Jaafar H Jaafar NRN Baharudin A Ibrahim N. The Relationship between Internet Addiction, Internet Gaming and Anxiety among Medical Students in a Malaysian Public University during COVID-19 Pandemic. Int J Environ Res Public Health. (2021) 18:11870. 10.3390/ijerph182211870 34831627 PMC8618673

[32] He T Huang CQ Li M Zhou YQ Li SH. Social participation of the elderly in China: The roles of conventional media, digital access and social media engagement. Telemat Inform. (2020) 48101347. 10.1016/j.tele.2020.101347

[33] Rollan VOD. Political participation of elderly people: Beyond voting. Aposta-Rev De Ciencias Soc. (2018) 79:164–80.

[34] Hsu HC. Does social participation by the elderly reduce mortality and cognitive impairment? Aging Mental Health. (2007) 11:699–707. 10.1080/13607860701366335 18074257

[35] Hao G Bishwajit G Tang SF Nie CP Ji L Huang R. Social participation and perceived depression among elderly population in South Africa. Clin Interv Aging. (2017) 12:971–6. 10.2147/CIA.S137993 28694690 PMC5491569

[36] Cai S. Does social participation improve cognitive abilities of the elderly? J Popul Econ. (2022) 35:591–619. 10.1007/s00148-020-00817-y

[37] Carstensen LL. Social and emotional patterns in adulthood - support for socioemotional selectivity theory. Psychol Aging. (1992) 7:331–8. 10.1037/0882-7974.7.3.331 1388852

[38] Marziali E. Gerontechnology: Research and practice in technology and aging. Canad J Aging. (2006) 25:237–8. 10.1353/cja.2006.0039

[39] Hlebec V. Social consequences of Internet use: access, involvement, and interaction. New Media Soc. (2004) 6:429–31.23428825

[40] Boekel L Peek ST Luijkx KG. Diversity in older adults' use of the internet: identifying subgroups through latent class analysis. J Med Internet Res. (2017) 19:e180. 10.2196/jmir.6853 28539302 PMC5463053

[41] Zheng ZH Chen H Yang L. Transfer of Promotion Effects on Elderly Health with Age: From Physical Environment to Interpersonal Environment and Social Participation. Int J Environ Res Public Health. (2019) 16:2794. 10.3390/ijerph16152794 31387307 PMC6696029

[42] Xinxin MA. Social participation and self-reported health in China: evidence from Chinese middle-aged and elderly adults. Int J Soc Econ. (2021) 48:85–103. 10.1108/IJSE-03-2020-0139

[43] Benyamini Y Leventhal H Leventhal EA. Self-rated oral health as an independent predictor of self-rated general health, self-esteem and life satisfaction. Soc Sci Med. (2004) 59:1109–16. 10.1016/j.socscimed.2003.12.021 15186909

[44] Meng QQ Xie Z Zhang TH. A single-item self-rated health measure correlates with objective health status in the elderly: a survey in suburban Beijing. Front Public Health. (2014) 2:27. 10.3389/fpubh.2014.00027 24783187 PMC3989711

[45] Chanda S Mishra R. Impact of transition in work status and social participation on cognitive performance among elderly in India. BMC Geriatr. (2019) 19:1–10. 10.1186/s12877-019-1261-5 31510923 PMC6737668

[46] Lake RL Huckfeldt R. Social capital, social networks, and political participation. Polit Psychol. (1998) 19:567–84. 10.1111/0162-895X.0011820229225

[47] Hyyppa MT Maki J Alanen E Impivaara O Aromaa A. Long-term stability of social participation. Soc Indic Res. (2008) 88:389–96. 10.1007/s11205-007-9199-y

[48] Baron RM Kenny DA. The moderator mediator variable distinction in social psychological-research - conceptual, strategic, and statistical considerations. J Pers Soc Psychol. (1986) 51:1173–82. 10.1037/0022-3514.51.6.1173 3806354

[49] Yang HL Zhang S Cheng SM Li ZY Wu YY Zhang SQ. A study on the impact of Internet use on depression among Chinese older people under the perspective of social participation. BMC Geriatr. (2022) 22:1–11. 10.1186/s12877-022-03359-y 35999498 PMC9398499

[50] Lai CKY Arthur DG Chau WWH. Implication of Internet growth on enhancing health of disadvantaged groups in China: a global perspective. J Clin Nurs. (2004) 13:68–73. 10.1111/j.1365-2702.2004.01046.x 15724821

[51] Boase J. The consequences of personal networks for internet use in rural areas. Am Behav Sci. (2010) 53:1257–67. 10.1177/0002764210361681

[52] Ge DD Chu J Zhou CC Qian YY Zhang L Sun L. Rural-urban difference in the use of annual physical examination among seniors in Shandong, China: a cross-sectional study. Int J Equity Health. (2017) 16:1–9. 10.1186/s12939-017-0585-z 28535772 PMC5442864

[53] Zhang J Li D Gao JM. Health Disparities between the Rural and Urban Elderly in China: A Cross-Sectional Study. Int J Environ Res Public Health. (2021) 18:8056. 10.3390/ijerph18158056 34360346 PMC8345474

[54] Gracia E Herrero J. Internet Use and Self-Rated Health Among Older People: A National Survey. J Med Internet Res. (2009) 11:e1311. 10.2196/jmir.1311 19955041 PMC2802559

[55] Liang X Xiong FX Xie FT. The effect of smartphones on the self-rated health levels of the elderly. BMC Public Health. (2022) 22:1–12. 10.1186/s12889-022-12952-0 35292010 PMC8925210

[56] Lelkes O. Happier and less isolated: internet use in old age. J Poverty Soc Justice. (2013) 21:33–46. 10.1332/175982713X664047

[57] Srivastava SK Panigrahi PK. Social participation among the elderly: Moderated mediation model of information and communication technology (ICT). Commun Assoc Inf Syst. (2019) 44:698–717. 10.17705/1CAIS.04433

